# Development of a rapid point-of-care dengue virus type 2 infection diagnostic assay using recombinase polymerase amplification and lateral flow device

**DOI:** 10.3389/fcimb.2025.1578549

**Published:** 2025-05-14

**Authors:** Meagan A. Prescott, Myra T. Koesdjojo, David T. Mandrell, Manoj K. Pastey

**Affiliations:** ^1^ Department of Biomedical Sciences, College of Veterinary Medicine, Oregon State University, Corvallis, OR, United States; ^2^ Department of Microbiology, College of Science, Oregon State University, Corvallis, OR, United States; ^3^ Custom Integration Services, KTM Research LLC, Hubbard, OR, United States

**Keywords:** dengue virus, real-time reverse transcription recombinase polymerase amplification, lateral flow detection (LFD), low-resource settings, point-of-care diagnostics

## Abstract

**Introduction:**

Dengue virus (DENV) is the most rapidly spreading arbovirus globally, with over half of the world’s population at risk of infection. Early and rapid detection is crucial to ensure timely patient care, reduce healthcare burden, and prevent severe disease progression. However, conventional nucleic acid amplification techniques are often unsuitable for low-resource settings due to their equipment and procedural demands.

**Methods:**

We evaluated a real-time reverse transcription recombinase polymerase amplification (RT-RPA) assay for the sensitive and specific detection of DENV serotype 2 (DENV2). The assay was tested using both Twista fluorometer and lateral flow detection (LFD) formats. Analytical sensitivity was determined by probit regression, while specificity was assessed against unrelated viruses and other flaviviruses. Clinical validation was performed using serum, cell culture, and FTA® card samples. Assay robustness was evaluated under varying temperatures and after freeze-thaw cycles.

**Results:**

The RT-RPA assay reliably amplified DENV2 at concentrations as low as 50 copies per reaction, with LOD₉₅ estimated at 38.48 copies (Twista) and 50.37 copies (LFD). No cross-reactivity was observed with respiratory syncytial virus, influenza, rabbit herpes virus, West Nile virus, or other DENV serotypes (DENV1, DENV3, DENV4). The assay successfully detected multiple DENV2 strains and maintained performance across 33°C–40°C and after repeated freeze-thaw cycles. RNA extracted from FTA® cards was successfully amplified. Clinical validation confirmed accurate detection in serum and cell culture samples, while DENV3-positive blood samples tested negative, reinforcing specificity.

**Discussion:**

The RT-RPA/LFD assay offers a rapid, sensitive, and specific tool for DENV2 detection, compatible with low-resource and field-based settings. Its simplicity, robustness, and portability make it a promising approach for point-of-care diagnostics and outbreak surveillance in endemic regions.

## 1 Introduction

Dengue virus (DENV) is a significant and widely distributed member of the *Flaviviridae* family. The World Health Organization (WHO) classifies dengue as a global pandemic threat and the most critical mosquito-borne viral disease worldwide (WHO, 2012). Dengue is endemic in over 100 countries across tropical and subtropical regions, with sporadic outbreaks occurring in additional locations worldwide (WHO, 2012). Since early 2023, ongoing transmission combined with an unexpected surge in dengue cases has resulted in a historic high of over 6.5 million cases and more than 7,300 dengue-related deaths (WHO, 2025). Annually, an estimated 100 to 390 million new infections occur, and alarmingly, the incidence of dengue has increased 30-fold over the last five decades (Malavige et al., 2023; Bhatt et al., 2013; WHO, 2012). Dengue outbreaks impose a substantial burden on healthcare systems and economies while contributing to morbidity and mortality. Additionally, disease underreporting and misclassification remain prevalent challenges (Malavige et al., 2023; Bhatt et al., 2013; Shepard et al., 2013; Edillo et al., 2015; Gulland, 2013; Salmon-Mulanovich et al., 2015; Shepard et al., 2014; Undurraga et al., 2015; Wichmann et al., 2011; Beatty et al., 2011). Managing dengue infections is increasingly complex, as symptoms often resemble those of other endemic diseases (Potts and Rothman, 2008). Consequently, rapid and sensitive diagnostic tests that can be administered early in disease progression are crucial for effective case management.

Dengue infection manifests along a spectrum from asymptomatic cases to severe disease. While most infections present as mild dengue fever, some progress to severe dengue hemorrhagic fever or dengue shock syndrome (Whitehorn and Simmons, 2011). The virus consists of four serotypes (DENV1-4); primary infection with one serotype confers lifelong immunity to that serotype but increases the risk of severe disease upon secondary infection with a different serotype due to antibody-dependent enhancement. Additionally, the sequence of serotype infections influences disease severity (Guzman et al., 2013; Fried et al., 2010; Endy et al., 2004; Ananta Preecha et al., 2005; de Araújo et al., 2009; Gibbons et al., 2007; Halstead, 2006; Sangkawibha et al., 1984; Thomas et al., 2008; Vaughn et al., 2000; Trivedi and Chakravarty, 2022).

Accurate and efficient DENV diagnosis is essential for surveillance, early detection, case confirmation, and differentiation from other diseases (WHO, 2009). Various diagnostic methods are available depending on the stage of infection. During the early phase (4–5 days post-infection), direct virus detection is possible, whereas serological methods are required later when the virus is no longer detectable. In general, high-confidence tests tend to be complex and time-consuming, limiting their widespread use, while rapid serological tests (based on antibody detection) often lack sensitivity or are unsuitable for early diagnosis (WHO, 2009).

Viral isolation, the gold standard for DENV detection, is impractical in clinical settings due to its time-intensive nature (Yamada et al., 2002; Medina et al., 2012). Serology-based assays, including IgM/IgG enzyme-linked immunosorbent assays (ELISA) and nonstructural protein 1 (NS1) antigen detection, are widely used due to their speed, but they exhibit reduced sensitivity and specificity (Hunsperger et al., 2014; Peeling et al., 2010; Hunsperger et al., 2009; Fernández and Vázquez, 1990; Moi et al., 2013; Paranavitane et al., 2014; Blacksell et al., 2012; Felix et al., 2012; Saito et al., 2015). Nucleic acid-based methods enable direct viral detection within a short timeframe. Real-time reverse transcription PCR (RT-PCR) has been employed for many years (Lanciotti et al., 1992; Johnson et al., 2005; Najioullah et al., 2014; Santiago et al., 2013; Zhao et al., 2024), but its complexity and resource requirements make it unsuitable for point-of-care testing. Recently, isothermal amplification techniques, such as nucleic acid sequence-based amplification (NASBA) (Wu et al., 2001) and reverse transcription loop-mediated isothermal amplification (RT-LAMP) (Sahni et al., 2013; Teoh et al., 2015), have been explored as alternatives that retain sensitivity while improving accessibility.

Recombinase polymerase amplification (RPA) is a promising isothermal amplification technique that offers advantages over traditional PCR and other isothermal methods. RPA is an extremely rapid (≤20 minutes) DNA amplification method that relies on three key enzymes: recombinases, which facilitate primer-target binding; single-stranded binding proteins (SSB), which stabilize the displaced DNA strands; and a strand-displacing polymerase, which enables DNA synthesis (Piepenburg et al., 2006). When coupled with reverse transcriptase (RT), RPA allows for RNA-to-cDNA conversion and subsequent amplification in a single-tube reaction (Bonnet et al., 2022). Detection of amplification products can be achieved via real-time fluorescence or lateral flow devices (LFD) (Boehringer and O’Farrell, 2021; Kakkar et al., 2024). In real-time RT-RPA, a fluorescent exo-probe is included in the reaction mix, whereas LFD-based detection utilizes 5’-modified primers and a colorimetric signal upon application to the LFD. This combination enables rapid, sensitive, and equipment-free detection of specific viral targets, making it particularly well-suited for low-resource settings, outbreak response, and decentralized testing. In resource-limited regions where laboratory infrastructure is minimal, this dual-format strategy provides both flexibility and accessibility, allowing critical diagnostic testing to be brought closer to the point of care.

RT-RPA combined with LFD has been successfully used for rapid detection of various viruses, including SARS-CoV-2, influenza, and hepatitis B (Zhao et al., 2024). While pan-DENV assays exist, few target specific serotypes like DENV2, which is often linked to severe dengue in regions such as India and the Americas. Serotype-specific detection offers greater clinical and epidemiological value. To address this gap, we developed a sensitive, field-deployable RT-RPA/LFD assay for DENV2, aiming to improve rapid diagnosis and support dengue control efforts.

## 2 Materials and methods

### 2.1 Virus and RNA

DENV2 laboratory strains were grown in Vero cells, harvested, and stored at -80°C. The typical dengue virus titer from Vero cells were 10_7_ PFU/mL. The laboratory strains of DENV2 used in this study were NGC, S221, and TH-36. RNA collected from these laboratory strains was extracted for RT-RPA using the NucleoSpin RNA isolation kit (Macherey-Nagel, Düren, Germany). Extractions were performed following the supplied protocols and guidelines. RNA was aliquoted into single-use tubes and stored at -80°C. Samples were processed in a Biosafety Level 2 (BSL-2) facility, ensuring safe handling of DENV2 and preventing exposure to infectious agents.

### 2.2 Assay design overview

#### 2.2.1 Primers and probe design

**Figure 1:**
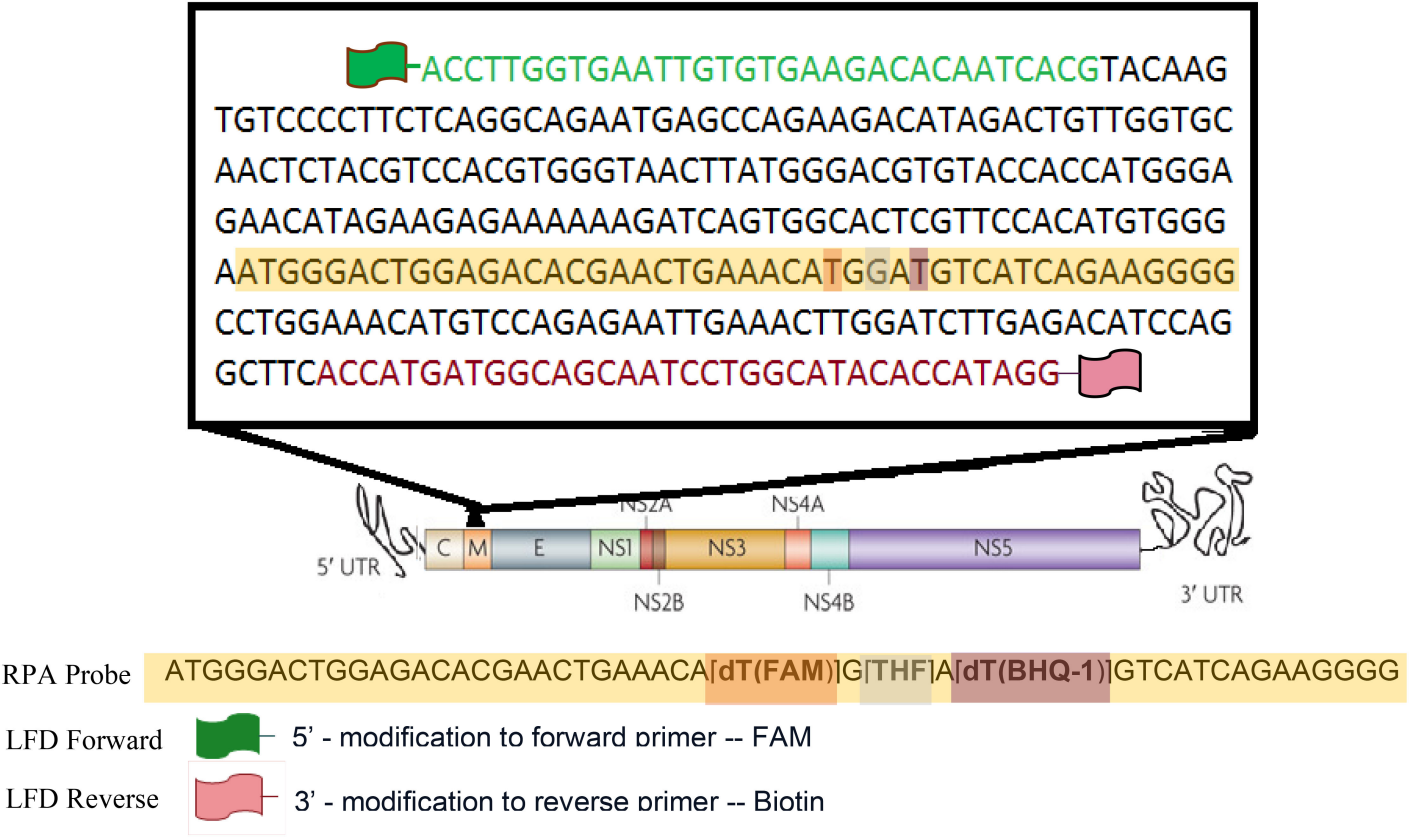
Schematic diagram of M gene showing RPA and LFD primers and probe used.

Primers compatible with the RPA reaction were designed to specifically amplify DENV2 (**Table 1**; **Figure 1**). The primer set was designed to amplify a 316 bp segment of the DENV2 viral genome corresponding to a portion of the structural membrane (M) protein (**Figure 1**). The M protein gene of DENV-2 is relatively conserved among DENV-2 isolates and serves as a good target for RPA due to its moderate length and role in viral assembly. A multiple sequence alignment of DENV-2 strains was performed to confirm minimal variation in the primer and probe binding sites.

**Table 1:** RPA and LFD primers and probe used for the rapid detection of DENV2.

Name	Sequence 5’-3’	GenBank Accession
Real-time		
DENV2_3F	ACCTTGGTGAATTGTGTGAAGACACAATCACG	PP269861.1
DENV2_1R	CCTATGGTGTATGCCAGGATTGCTGCCATTATGGT	PP269937.1
DENV2_Probe	ATGGGACTGGAGACACGAACTGAAACA[**dT(FAM**)]**G[THF**] A[**dT(BHQ-1**)]GTCATCAGAAGGGG	PP269903.1
LFD		
DENV2_3F_LFD	**FAM**-ACCTTGGTGAATTGTGTGAAGACACAATCACG	PP269861.1
DENV2_1R_LFD	**Biotin**-CCTATGGTGTATGCCAGGATTGCTGCCATTA TGGT	PP269937.1

Bold text within the sequence denotes modifications made to the oligonucleotides. dT(FAM): thymidine nucleotide bearing fluorescein, THF: tetrahydrofuran spacer replacing G nucleotide, and dT(BHQ-1): thymidine nucleotide bearing the black hole quencher 1.

In addition, *in silico* analyses (e.g., BLAST alignment) and experimental cross-reactivity testing were conducted using unrelated viral templates, including respiratory syncytial virus, influenza (PR/8/34), and rabbit herpes virus, as well as several related flavivirus members such as West Nile virus and RNA from the other DENV serotypes.

**Figure 2:**
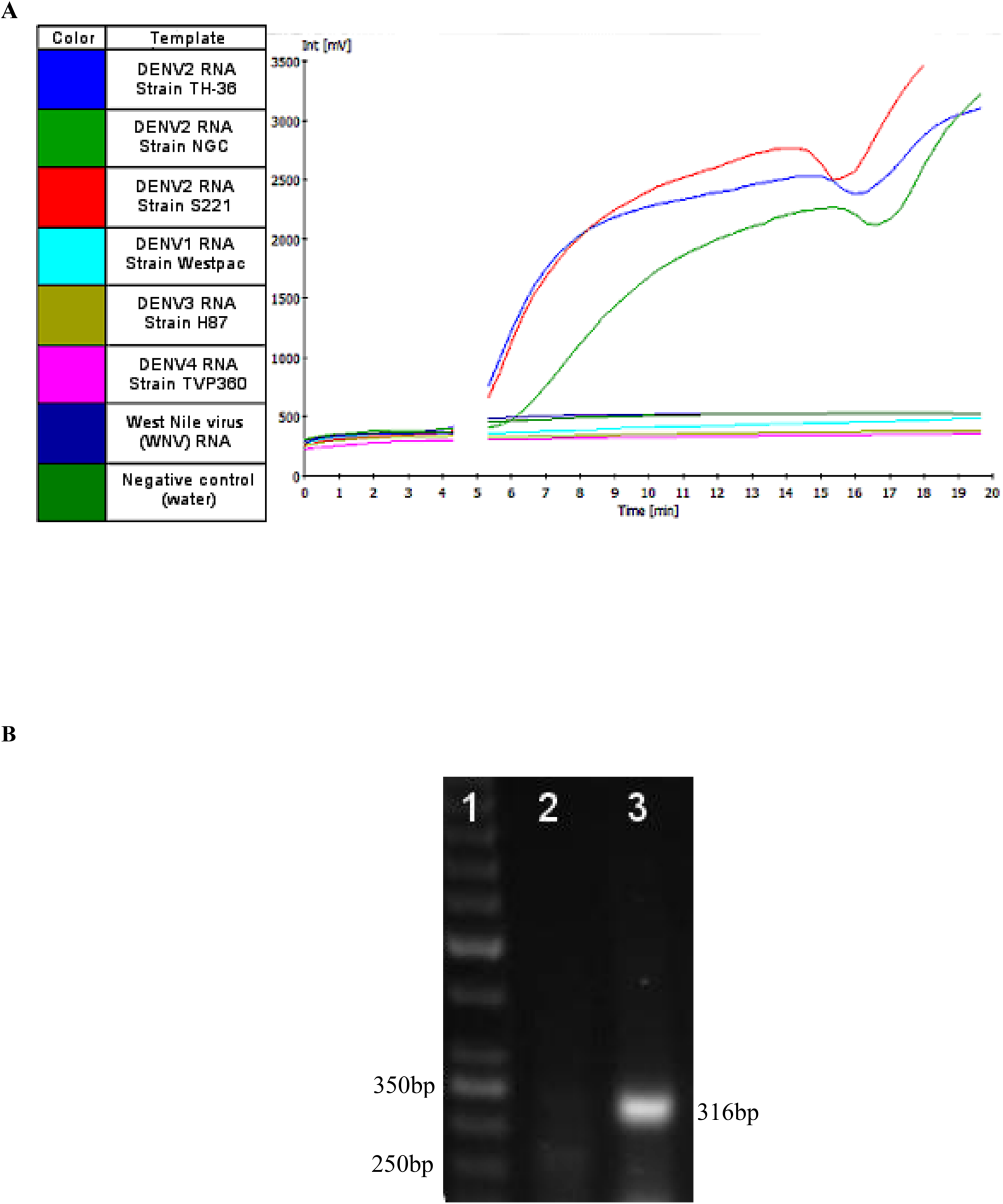
**(A)** A representative image of real-time RT-RPA amplification products using the Twista fluorometer. The graph depicts the fluorescent signal for each template used in the RT-RPA reactions and the key to the left indicates the template each color represents. At approximately five minutes into run time a required Magnesium acetate (280 nM) adding step, to initiate the RPA reaction, was performed resulting in signal not being generated for the duration of that step (gap in the graph). **(B)** RPA amplification of Dengue 2 M gene. The numbers in the left margin indicate selected bands of the DNA marker, and the estimated amplified product size is indicated at 316 bp in the right margin. Lane 1 is a 50bp DNA ladder (Thermo Scientific™), lane 2 is the no-template control (water as template). Lanes 3 represent RPA amplification product from 10 copies of DENV-2 genome per reaction at 37°C.

Real-time RT-RPA generated a clear fluorescence signal using standard primers and a FAM-labeled exo probe (**Figure 2A**). Signal generation occurred upon probe hybridization and cleavage by Exonuclease III at the tetrahydrofuran (THF) site, separating the fluorophore from the quencher. This enabled specific and real-time detection of DENV2 amplification.

#### 2.2.2 Real-time reverse transcription recombinase polymerase amplification

DENV2 genome amplification was performed using the TwistAmp exo RT kit (TwistDx, Ltd., Cambridge, United Kingdom) (Twist, 2018). Depending on the detection format, appropriate primers were used (**Table 1**; **Figure 1**). The kit contained a lyophilized pellet with reaction enzymes, including the RT enzyme necessary for generating complementary DNA (cDNA) from an RNA template, premeasured and distributed by the manufacturer.

The reaction pellet was dissolved following the manufacturer’s instructions using a mix of rehydration buffer, diluted template, appropriate primer/probe sets, and water to a final volume of 47.5 μl. Magnesium acetate (280 nM) was used to initiate the RPA reaction, bringing the final reaction volume to 50 µl. The mixture contained 0.5 µM forward and reverse primers and 0.2 µM probe. A non-template control (water with no DNA) and a positive control using NGC DENV2 were included in each run.

#### 2.2.3 Limit of detection

**Figure 3:**
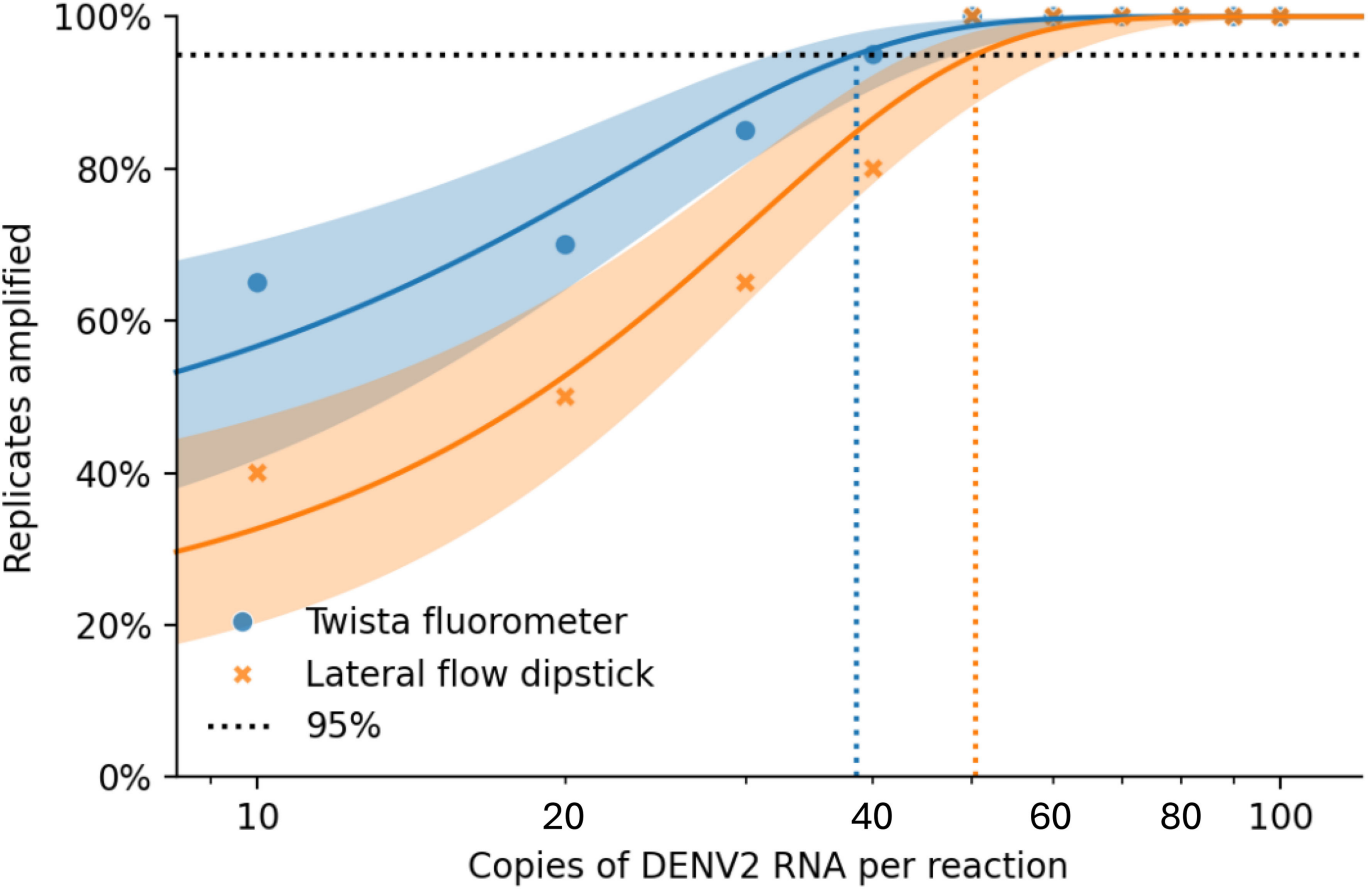
Combined Probit regression plot comparing Twista (fluorescence detection) and LFD (lateral flow): • Solid blue curve: Twista with 95% CI • Solid Orange curve: LFD with 95% CI • Blue circles: Observed Twista results • Orange cross: Observed LFD results • Dashed vertical lines show the estimated LoD at 95% detection for each method: o Twista ≈ 38.48 copies/reaction o LFD ≈ 50.37 copies/reaction.

Accurate assessment of nucleic acid copy number is critical for determining the analytical sensitivity of molecular assays, such as RT-RPA. It allows for precise quantification of the target RNA or DNA, enabling the evaluation of detection limits (e.g., LoD95) and ensuring reproducibility across experiments. For assessing genome copy numbers, a ten-fold serial dilution series of DENV2 (NGC strain) RNA was prepared. Starting from a stock solution of known concentration, dilutions were made in nuclease-free water (or an appropriate buffer) to generate final concentrations of 100, 90, 80, 70, 60, 50, 40, 30, 20, and 10 copies per reaction. Each dilution was aliquoted and tested in 20 replicate reactions to determine the detection rate at each concentration (**Table 2**; **Figure 3**).

**Table 2:** Detection of DENV2 RNA by RT-RPA across different copy numbers.

Copies of DENV2 per reaction	Positive amplification	Positive amplification
	(Twista fluorometer)	(LFD)
100	20/20	20/20
90	20/20	20/20
80	20/20	20/20
70	20/20	20/20
60	20/20	20/20
50	20/20	20/20
40	19/20	16/20
30	17/20	13/20
20	14/20	10/20
10	13/20	8/20

Table shows the number of positive results out of 20 replicates for each input concentration using both the Twista fluorometer and LFD.

Additionally, serial dilutions of positive control plasmids, pTM-M (containing the M gene), were included. A 316 bp fragment (nt. 524–840; GenBank accession no. PP269861.1) of the membrane glycoprotein region was cloned into the pTM vector. The recombinant plasmids used as standard nucleic acids were quantified using a NanoDrop ND-1000 spectrophotometer, and the DNA copy number was calculated using the following formula:


$$DNAcopynumber(copies/\mu L)\frac{6.022x10^{23}xplasmidconcentration(ng/\mu L)}{plasmidmolecularweight(g/mol)x10^{9}}$$

The recombinant plasmid was serially diluted from 10¹°C to 10 copies/μL and stored at −20°C until use. The reaction was conducted at 40 °C for 20 minutes, and amplification products were visualized using either LFD or real-time fluorescence detection (data not shown).

#### 2.2.4 Confirmation of RT-RPA amplified DENV2 DNA product

To confirm the correct DNA product was amplified, Sanger sequencing was performed at the Center for Genome Research and Biocomputing at Oregon State University. Following amplification, products were purified using a GeneJET DNA purification kit (Thermo Scientific, Waltham, MA, USA) and observed via gel electrophoresis on a 1.5% agarose gel stained with SYBR Safe (Life Technologies, Carlsbad, CA, USA) (**Figure 2B**). DNA band was visualized under an LED blue light transilluminator, and the 316 bp product (**Figure 2B**) was excised and purified using the GeneJet kit. Sequencing results were aligned to the predicted DENV2 sequence using the EMBOSS Water pairwise alignment tool.

#### 2.2.5 Detection of amplified product

##### 2.2.5.1 Fluorescence detection of real time RT-RPA

**Figure 4:**
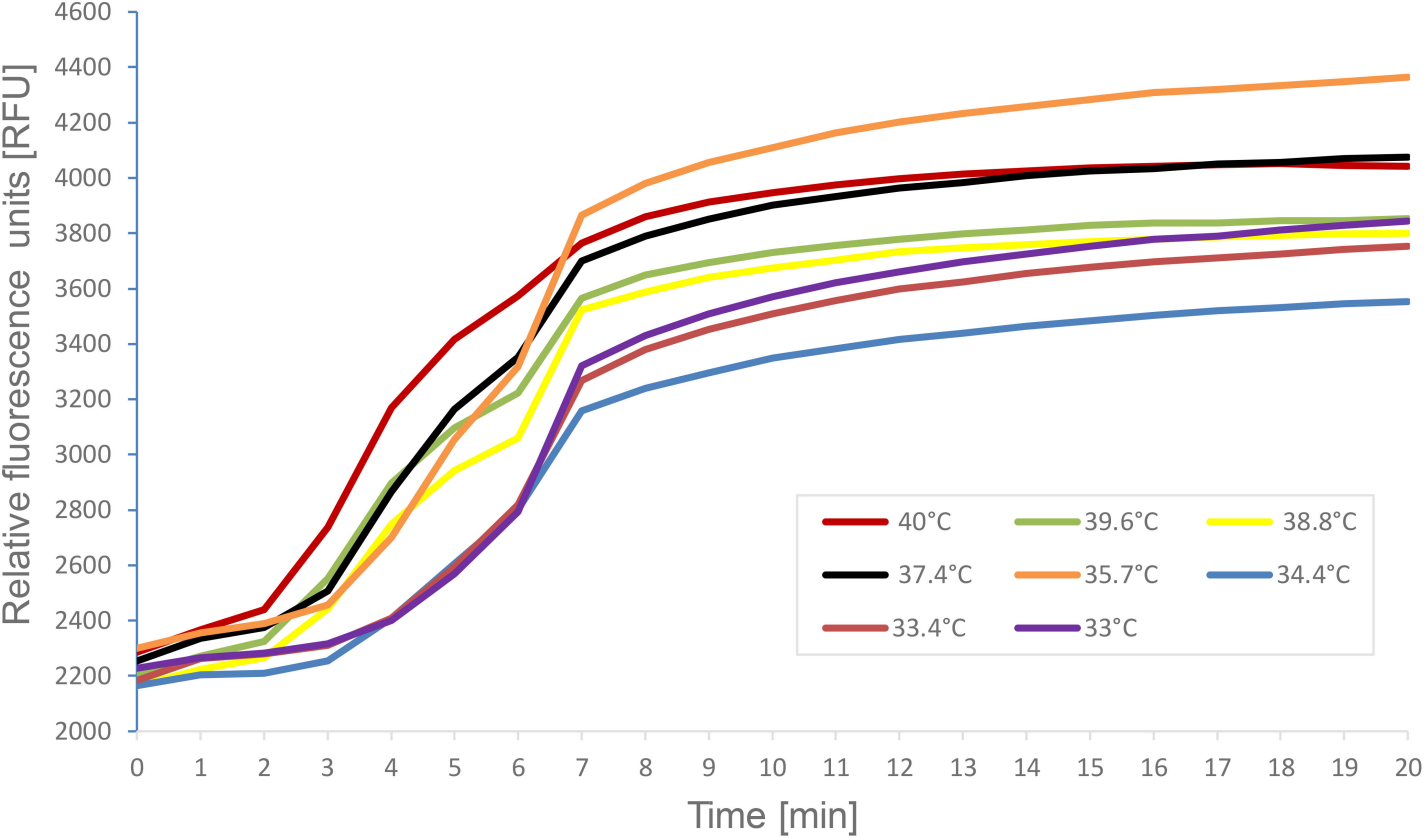
A representative image of the reaction temperature range for DENV2 specific real-time RT-RPA amplification products. Fluorescence measurements were made using the CFX96 Touch™ Real-Time PCR Detection System. The graph depicts the fluorescent signal for each of RT- RPA reactions which varied only in the temperature conditions. The key indicates the color on the graph that represents the temperature each RT- RPA reaction was run under.

Real-time RT-RPA was achieved using the TwistAmp exo RT kit (TwistDx) (Twist, 2018). Reactions containing the basic RPA primers with the DENV2-specific exo-probe allowed real-time visualization of amplification products via fluorescence signal monitoring. Amplification was monitored using the portable Twista real-time fluorometer (TwistDx) (**Figure 2A**). For experiments involving temperature gradients, the Bio-Rad CFX96 Touch™ Real-Time PCR Detection System (Bio-Rad Laboratories, Hercules, CA, USA) was used (**Figure 4**). The one-step RT-RPA reaction is completed within 20 minutes.

##### 2.2.5.2 Lateral flow device detection of RT-RPA products

**Figure 5:**
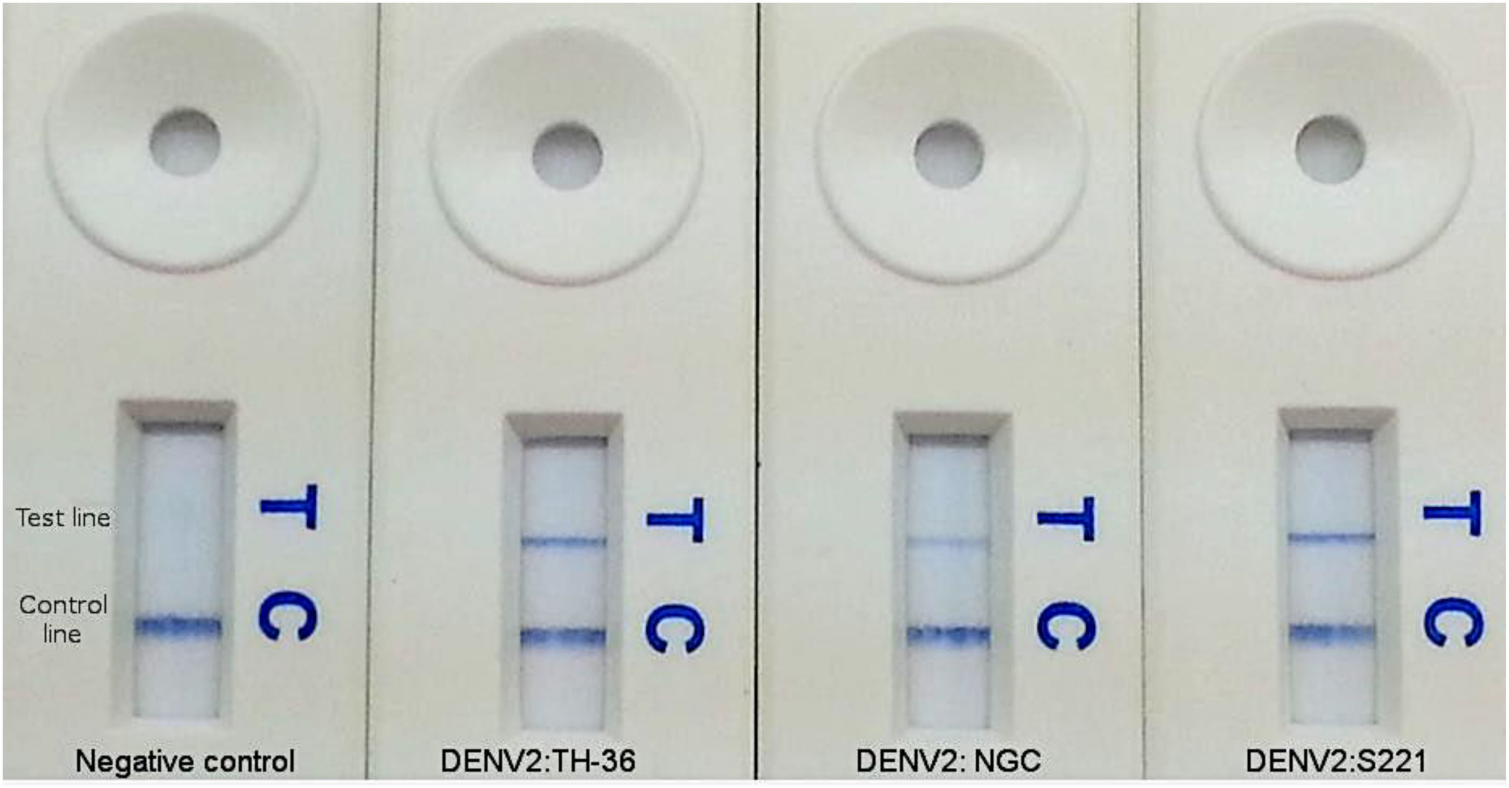
Amplification products for DENV2 RT-RPA assay visualized by LFD. Colorimetric signal at the test line (T) indicates positive amplification. Signal at control line (C) indicates a valid test.

**Figure 6:**
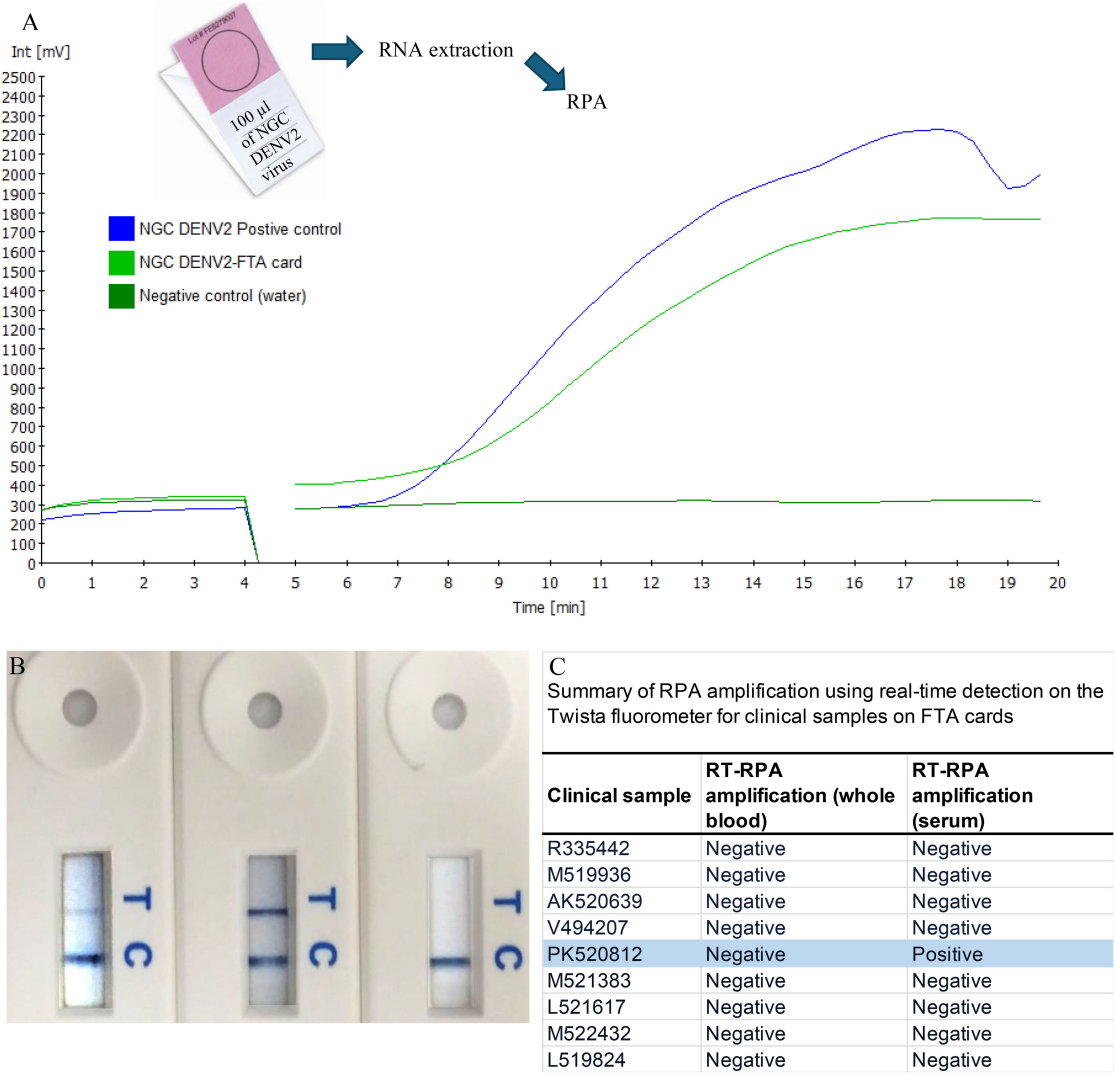
Amplification of samples from FTA cards. RNA collected from FTA^®^ cards spiked with NGC DENV2 was amplified by our RT-RPA assay and visualized using both real-time **(A)** and LFD formats **(B)**. Following this spiked trial, the clinical samples obtained from patients in India were tested **(C)**. RNA from both whole blood and serum FTA^®^ card samples were analyzed by RT-RPA specific to DENV2. For each of the replicates tested, one of the patient serum samples (PK520812s) was amplified by RT-RPA measured by both the real time and LFD formats **(C)**.

Rapid detection of the DENV2 genome was also achieved by LFD. DENV2 genome amplification was conducted using the TwistAmp exo kit (TwistDx) (Twist, 2018), with the 5’ modified primers replacing standard primers in the reaction mix. The optimal concentration of labeled primers was determined to be 1.25 μM. The end products, labeled with biotin and FAM, were diluted in buffer and applied to a PCRD-2 LFD (Forsite Diagnostics, UK) (**Figures 5**, **6B**). In brief, 5 μL of RPA products were added into 100 μL of sample buffer and mixed thoroughly. Then, 60 μL of the mixed solution was dispensed onto the lateral-flow dipsticks and results were recorded until the positive control line was visualized. A colorimetric signal indicated the presence of doubly labeled DNA at the test line (T-line) via immobilized anti-FAM and anti-biotin antibodies, with a control line (C-line) ensuring proper flow. Positive results were indicated by two lines (C-line and T-line), while negative results showed only the C-line. Tests without visible lines were considered faulty and unusable. The LFD detection format added no more than 10 minutes to the reaction time and enabled product visualization without the need for costly exo-probes or real-time detection equipment.

The lateral flow device used in this study was optimized to ensure absolute accuracy by eliminating the possibility of false positives and false negatives. The following measures were implemented: a. Every test result was verified using quantitative PCR (qPCR); b. Each sample was tested in at least three independent replicates to confirm consistency; c. The LFD assay included built-in internal controls to ensure proper test functionality; d. Key reaction parameters such as incubation time, reaction temperature, and primer concentration were systematically optimized; e. A stringent threshold for positive detection was established based on calibration with standard reference materials, ensuring that only true positives were reported, f. To assess potential cross-reactivity, the LFD assay was tested against respiratory syncytial virus (RSV), influenza (PR/8/34), and rabbit herpes virus, as well as several related flavivirus members; West Nile virus (WNV) and RNA from the other DENV serotypes (**Table 3**).

**Table 3:** Summary of DENV2 RPA amplification using real-time detection on the Twista fluorometer and LFD.

Template	RT-RPA amplification	LFD detection
Laboratory samples		
DENV2: strain NGC	Positive	Positive
DENV2: strain TH-36	Positive	Positive
DENV2: strain S221	Positive	Positive
DENV1: strain Westpac	Negative	Negative
DENV1: strain TH-SMAN	Negative	Negative
DENV3: Strain H87	Negative	Negative
DENV4: Strain H241	Negative	Negative
DENV4: Strain TVP 360	Negative	Negative
WNV	Negative	Negative
RSV	Negative	Negative
Influenza	Negative	Negative
Herpes virus	Negative	Negative
Tissue culture negative	Negative	Negative
negative control (water)	Negative	Negative
Clinical samples		
DENV_4	Positive	Positive
DENV_5	Positive	Positive
HTV 201	Negative	Negative
HTV 277	Negative	Negative
HTV 337	Negative	Negative

The source of the template used in the RT-RPA reaction is indicated in the template column and the RT-RPA amplification column indicates whether amplification was observed. For LFD, positive results were indicated by two lines (C-line and T-line), while negative results showed only the C-line.

### 2.3 Clinical sample collection and ethics compliance

#### 2.3.1 Ethics statement

Serum samples for clinical performance studies were obtained through the Dengue Surveillance System sample collection under guidelines approved by the Medical Faculty University Padjajaran (Bandung, Indonesia), M.S. Ramaiah Medical College Hospital (Bangalore, India) and Oregon State University (OSU) institutional review boards (IRB Approval #8053 on May 09, 2021). Patients did not provide verbal or written consent for this study. Samples were de-linked from patient identifiers according to IRB protocols.

A total of five acute serum samples from clinically suspected DENV-infected patients (average age: 20.3 years) were collected between 2022–2023 at Hospital Dokter Hasan Sadikin (Bandung, Indonesia) (**Table 3**). The RT-RPA assay was performed on a subset of these clinical samples at the Medical Faculty University Padjajaran (Bandung, Indonesia). Two samples (DENV_4, DENV_5) represent RNA extracted from cultured DENV2-positive patients confirmed by Lanciotti (RT-PCR) (Lanciotti et al., 1992). In addition, RNA from three patient serum samples (HTV093, HTV277, and HTV201) suspected to be DENV positive but previously unconfirmed were extracted using a Qiagen RNA extraction kit and tested using the DENV2-specific RT-RPA real-time assay (**Table 3**).

#### 2.3.2 Patient sample collection on Watman FTA^®^ cards

The purpose of using Flinders Technology Associates (FTA) cards for sample collection is two-fold. First, people in developing countries who live in remote villages may not be able to travel to city hospitals and the FTA card may benefit them if they are willing to send a finger pin-prick blood sample on FTA card for diagnosis. Secondly, surveillance of mosquitoes for dengue from different locations can be accomplished as the FTA card preserves RNA samples for a considerable time until processing.

Sample collection using Whatman FTA^®^ technology (GE Healthcare Bio- Sciences Corp., USA) was also tested with both real-time and LFD (**Figures 6A, B**). Initial screens were prepared using spiked samples that contained 100 µl of DENV2 (NGC) virus from cell culture and applied to Indicating FTA^®^ Micro Cards. After drying for at least 2 hours, RNA was extracted. At M.S. Ramaiah Medical College Hospital, India, a total of nine acute serum samples were collected from febrile patients during 2022–2023, suspected of having dengue 0–5 days after onset of symptoms and whose average age was 18.5 years (**Figure 6C**). For each of the nine patients sampled, both whole blood and serum samples were collected and applied to FTA^®^ cards. These samples were confirmed by hospital personnel to have DENV infection by the NS1 based ELISA method (Dengue NS1 Ag Microlisa Kit, J.Mitra and Co Pvt. Ltd, India) but no information on serotype was available. Cards were stored for several months at room temperature followedby RNA extraction from the FTA^®^ cards by the Molecular Diagnostics Laboratory at Oregon State University. Sample preparation was accomplished using the Ambion RNA Rapid Extraction solution (Life Technologies) followed by isolation using the Ambion MagMAX 96 viral RNA isolation kit (Life Technologies). The undiluted RNA was used as template in the RT-RPA assay and reactions were run in duplicate.

### 2.4 Assessment of DENV2 specific RT-RPA assay reaction conditions and robustness

#### 2.4.1 Temperature tolerance

Reaction conditions were also examined for the RT-RPA assay. Using the real-time format, identical RT-RPA reactions were run under several different temperature conditions (**Figure 4**). The Twista reader is capable of holding the reactions at only a single temperature, therefore, the CFX thermocycler was used as a fluorometer so, samples could be subjected to a gradient ofconstant temperatures. Reactions contained 10–^2^ dilution of DENV2 (NGC) and the temperatures tested ranged from the manufacture’s recommended 40°C down to 33°C. As seen in **Figure 4**, each temperature tested resulted in amplification products, highlighting the adaptable nature of RPA amplification in regard to temperature.

#### 2.4.2 Carry-over and cross-contamination

To ensure the accuracy and reliability of the detection process, rigorous contamination control measures were implemented. Prior to opening the sample cap, the exterior surface was disinfected with 70% ethanol to eliminate potential contaminants. All handling and processing were conducted within a laminar flow hood to prevent environmental contamination. To evaluate possible cross-contamination of samples while performing the assay, a panel of DENV-1–4 was used at high positive (10^7^ Genome Copy Equivalent (GCE/mL) and below limit of detection (LOD)(10^1^ GCE/mL) concentrations, respectively. Samples below LOD are composed of normal human serum spiked with DENV to a concentration just below the LOD. Five replicates of the high positive and below LOD DENV concentrations were extracted using the Ambion MagMAX 96 viral RNA isolation kit (Life Technologies) and tested in alternating series. All negative samples tested negative (5/5) and all DENV positive samples tested positive for DENV (5/5). Several below LOD samples produced CT values >39 that may correspond to detection of DENV RNA; however, these results are not 100% reproducible (data not shown).

#### 2.4.3 Performance in fresh vs. frozen specimens

To assess the impact of temperature fluctuations on assay performance, DENV2-spiked human serum samples were tested following multiple freeze–thaw cycles. Moderate and low positive dilutions were prepared in triplicate, frozen at −80°C for 24 hours, and subjected to five consecutive freeze/thaw cycles. Once thawed, every sample was processed according to the assay protocol. Data showed a 100% qualitative agreement between the initial and post freeze/thaw cycle (1, 2, 3, 4, and 5) detection results (data not shown).

### 2.5 Statistical analysis

All experimental data were analyzed using appropriate statistical methods to assess the sensitivity, specificity, and reproducibility of the RT-RPA assay. The limit of detection (LOD95) was determined using probit regression analysis to estimate the viral copy number at which 95% of reactions yielded positive results (**Figure 3**). Specificity was evaluated by testing the assay against non-target viruses, and results were analyzed for cross-reactivity. Reproducibility was assessed by performing triplicate experiments across independent runs and calculating mean cycle threshold (Ct) values, standard deviations, and coefficients of variation. Clinical validation results were analyzed using positive percent agreement (PPA), negative percent agreement (NPA), and overall accuracy in comparison to reference methods. Data analysis was conducted using GraphPad Prism and R statistical software, with significance levels set at *p*< 0.05 where applicable.

## 3 Results

### 3.1 Sensitivity of DENV2 detection by real-time RT-RPA and LFD

#### 3.1.1 Analytical sensitivity

The sensitivity of the RT-RPA assay was evaluated using both real-time fluorescence (Twista fluorometer) and lateral flow device (LFD) formats. Although amplification was observed at input levels as low as 10–25 copies per reaction, only the 50-copy dilution consistently yielded positive results in 100% of replicates within the 20-minute reaction window. Similarly, the LFD format detected 50 copies per reaction across all replicates, though its sensitivity declined more rapidly at lower input concentrations compared to real-time detection (**Table 2**).

#### 3.1.2 Limit of detection by probit analysis

Probit regression analysis was performed to determine the 95% limit of detection (LoD_95_) for each detection method (**Figure 3**). The estimated LoD_95_ was approximately 38.48 copies/reaction for the Twista fluorometer and 50.37 copies/reaction for LFD. These findings demonstrate that real-time RT-RPA offers greater sensitivity, capable of detecting lower quantities of DENV2 RNA with high confidence.

#### 3.1.3 Strain coverage

The assay’s ability to detect multiple DENV2 strains was also assessed. RNA from each strain was diluted to 10² copies/reaction in nuclease-free water and tested in at least three replicates. All tested DENV2 strains were successfully amplified by the RT-RPA assay and detected by both the Twista fluorometer and LFD formats (**Figure 2A**; **Figure 5**; **Table 3**). Representative amplification results are shown in **Figures 2A** and **5**, confirming the assay’s broad detection capability across DENV2 variants.

### 3.2 Specificity of DENV2 detection by real-time RT-RPA and LFD

#### 3.2.1 Non-target testing

The specificity of the DENV2 RT-RPA assay was evaluated using both real-time fluorescence detection (Twista fluorometer) and LFD formats. A panel of non-target templates was tested in at least three replicates each to assess cross-reactivity. These included two negative controls—nuclease-free water and a tissue culture negative control—as well as a range of unrelated viral RNA templates: respiratory syncytial virus (RSV), influenza A virus (PR/8/34), and rabbit herpesvirus. In addition, several closely related flaviviruses were included: West Nile virus (WNV) and RNA from DENV serotypes 1, 3, and 4 (**Table 3**).

All non-target templates tested negative by both detection methods, indicating high specificity of the RT-RPA assay for DENV2.

#### 3.2.2 Product confirmation

Amplification reactions were performed using 0.05 µL of viral cDNA in a 50-µL reaction volume. Results of the specificity evaluation are summarized in **Table 1** and confirm that the selected primers are highly specific to the DENV2 genome. Amplicons from NGC samples were further analyzed by agarose gel electrophoresis (**Figure 2B**) and confirmed by sequencing. Sequencing results verified that the amplified products matched the expected DENV2 target region.

### 3.3 Detection methods of RT-RPA products

Amplification products were detected using two formats: real-time fluorescence via the Twista fluorometer and colorimetric visualization via a lateral flow device (LFD). Both detection methods yielded concordant results, with the Twista fluorometer and LFD producing identical outcomes across all tested samples. The LFD successfully generated visible positive signals for all DENV2 strains and negative results for non-DENV2 viral templates, including other flaviviruses and unrelated respiratory viruses (**Figure 2A**; **Figure 5**; **Table 3**). These results confirm the high specificity of the assay and its versatility across detection platforms.

LFD band intensity was observed to vary depending on reaction time, sample concentration, flow rate, and reagent interactions. Therefore, reaction conditions were standardized prior to sample testing to ensure consistency and reproducibility.

### 3.4 Assessment of DENV2-specific RT-RPA assay in clinical samples

#### 3.4.1 Detection in cell culture-derived samples

The RT-RPA assay was first evaluated using RNA from cell culture-derived samples. Two samples (DENV_4 and DENV_5), obtained from patients previously confirmed to be infected with DENV2, produced clear and consistent amplification using both real-time fluorescence (Twista fluorometer) and LFD formats (**Table 3**). These results confirmed the assay’s effectiveness in detecting DENV2 RNA under controlled sample conditions.

#### 3.4.2 Specificity evaluation in suspected clinical samples

Three suspected dengue-positive blood samples (HTV093, HTV277, and HTV201) failed to generate amplification with the DENV2-specific RT-RPA assay in either detection format (**Table 3**). Subsequent RT-PCR serotyping identified all three samples as DENV3, further validating the specificity of the RT-RPA assay for the DENV2 serotype and ruling out cross-reactivity with other dengue serotypes.

#### 3.4.3 Detection from FTA^®^ card samples

DENV2 RNA preserved on FTA^®^ cards was reliably detected using both Twista and LFD formats (**Figures 6A, B**). Clinical testing of FTA^®^ card samples from patients in India showed that one serum sample (PK520812s) yielded a positive amplification signal across all replicates with both detection methods (**Figure 6C**). In contrast, the matching whole blood sample from the same patient, as well as all other clinical samples collected on FTA^®^ cards, did not produce detectable signals.

### 3.5 Assessment of DENV2-specific RT-RPA assay reaction conditions and robustness

#### 3.5.1 Temperature tolerance

The RT-RPA assay demonstrated robust performance across a range of constant temperatures (33°C to 40°C) using a 10^-^² dilution of DENV2 RNA. Amplification was successful at all tested temperatures (**Figure 4**), confirming the assay’s adaptability to variable field or laboratory conditions.

#### 3.5.2 Carry-over and cross-contamination

To assess cross-contamination risk, DENV1–4 samples were tested in alternating high-positive (10^7^ GCE/mL) and below-LoD (10¹ GCE/mL) concentrations. All high-positive samples tested positive, and all low-level samples tested negative (5/5), confirming the effectiveness of contamination control measures. Sporadic late signals (Ct >39) in below-LoD samples were not reproducible.

#### 3.5.3 Freeze–thaw stability

DENV2-spiked serum samples subjected to five freeze–thaw cycles showed 100% agreement in detection across all replicates compared to fresh controls. This indicates the assay is resilient to temperature stress and suitable for use with stored or transported clinical specimens.

## 4 Discussion

Rapid and sensitive detection of DENV is critical to the disease management of a virus with such a prominent burden to human health (Kabir et al., 2021). The findings of this study highlight the potential of the real-time RT-RPA assay as a valuable tool for the rapid detection of DENV2. One of the primary strengths of the assay is its high sensitivity. The assay reliably amplified DENV2 at concentrations as low as 50 copies per reaction as detected by both Twista fluorometer and LFD, with probit regression estimating an LOD95 of approximately 38.48 copies per reaction for Twista fluorometer and 50.37 copies per reaction for LFD. This low detection limit is particularly promising for early diagnosis when viral loads might be low, thus facilitating prompt patient management and outbreak control. Equally important is the assay’s specificity. By rigorously testing against a range of non-DENV2 viral templates—including other flaviviruses and unrelated respiratory pathogens—the study confirmed that the selected primers exclusively target DENV2 RNA. This high specificity minimizes the risk of false positives, which is critical in areas where multiple flaviviruses co-circulate.

The dual-mode detection capability—real-time fluorescence and lateral flow device (LFD) readout—greatly enhances the assay’s versatility. The real-time format allows for quantitative assessments in a controlled laboratory environment, while the LFD format provides a rapid, equipment-free method suitable for point-of-care testing. This versatility is especially beneficial in resource-limited settings or during outbreak scenarios where rapid, on-site diagnostics can significantly impact public health responses.

Moreover, the assay demonstrated robust performance across a range of temperatures, from 33°C to 40°C, and after multiple freeze/thaw cycles, suggesting that it can accommodate variations in both environmental conditions and sample handling. This resilience is vital for field applications, where laboratory-grade environmental control may not be available. The ability to work effectively with samples stored on FTA^®^ cards further supports the potential for using this assay in remote or resource-constrained regions, facilitating safe sample collection and transport.

Validation of clinical samples demonstrated that the newly developed RT-RPA/LFD assay exhibits diagnostic performance comparable to that of the gold standard real-time RT-PCR assay for routine DENV detection (Wang and Gubler, 2018), but this method simplifies the instrumentation requirements and reduces the cost. Although methods like RT-RPA (Teoh et al., 2015) and LAMP (Dauner et al., 2015; Lopez-Jimena et al., 2018) enable rapid dengue diagnosis, the novel RT-RPA/LFD assay offers notable advantages. Its lateral-flow dipstick design allows results to be read by the naked eye in 10 mins, eliminating the need for specialized equipment and trained personnel. Additionally, operating at 37°C—using body heat for amplification—it is well-suited for field use and low-resource settings, unlike other methods that require precise temperature control and power-dependent instrumentation.

The combination of RT-RPA and LFD has been successfully applied for the rapid detection of SARS-CoV-2, influenza A and B, Coxsackievirus A6, and hepatitis B virus (Liu et al., 2021; Sun et al., 2019; Xie et al., 2021; Zhang et al., 2021; Zhao et al., 2024). Additionally, RT-RPA combined with LFD and CRISPR/Cas12a has been used to detect pan-DENV serotypes (Bhardwaj et al., 2025; Xi et al., 2019; Xiong et al., 2020). However, few studies have focused specifically on serotype-specific detection of DENV2 using this format. While pan-DENV detection confirms the presence of the virus, serotype-specific detection provides critical, actionable insights that improve patient management, inform public health interventions, support vaccine development, and enhance our understanding of dengue epidemiology.

Although all four dengue serotypes circulate globally, DENV2 is often considered the most prevalent serotype, particularly in regions such as India and the Americas, where it is associated with a higher proportion of severe dengue cases (Sirisena et al., 2021). Our work addresses this gap by developing a sensitive, field-deployable RT-RPA/LFD assay specifically targeting the DENV2 serotype. The implementation of this assay may facilitate rapid serotype-specific diagnosis and contribute to improved dengue control efforts in the current global health landscape.

While initial clinical evaluations, including tests on cell culture samples and FTA^®^ card-collected specimens, provided encouraging results, some limitations remain. For instance, the assay failed to amplify RNA from certain blood samples that were later identified as DENV3 infections, underscoring the assay’s high specificity for DENV2 but also pointing to the need for multiplexing capabilities if detection of all dengue serotypes is desired in a clinical diagnostic tool. Additionally, the relatively small dataset used for probit regression to estimate the LOD95 calls for future work involving larger numbers of clinical samples to refine the detection limits and ensure reproducibility across diverse conditions.

Future research should focus on developing a multiplex RT-RPA assay that can simultaneously detect multiple dengue serotypes or other co-circulating arboviruses, such as Zika and chikungunya. Larger-scale studies across diverse geographic regions and patient populations will be essential to validate the assay’s diagnostic performance and establish standardized protocols. An evaluation of the cost-effectiveness and scalability of the RT-RPA assay, compared to traditional RT-PCR methods, could facilitate its adoption in public health programs, and integrating data connectivity with the LFD format might streamline surveillance efforts and enhance data collection during outbreak monitoring.

In summary, the real-time RT-RPA assay for DENV2 exhibits considerable promise due to its high sensitivity, specificity, and operational versatility. Its adaptability to different detection formats and robustness under variable conditions position it as an attractive option for both laboratory and field-based diagnostics. With further validation, optimization, and expansion into multiplex formats, this assay could become a key tool in the early diagnosis and management of dengue outbreaks, ultimately contributing to improved patient care and more effective public health responses.

## Data Availability

The original contributions presented in the study are included in the article/supplementary material. Further inquiries can be directed to the corresponding author.
